# Association between ossification of the posterior longitudinal ligament and ossification of the nuchal ligament in the cervical spine

**DOI:** 10.1371/journal.pone.0224729

**Published:** 2019-11-06

**Authors:** Myung Sub Kim, Hee Jin Park, So Yeon Lee, Ji Na Kim

**Affiliations:** Department of Radiology, Kangbuk Samsung Hospital, Sungkyunkwan University School of Medicine, Seoul, Republic of Korea; Ohio State University, UNITED STATES

## Abstract

**Objectives:**

To investigate the association between ossification of the posterior longitudinal ligament (OPLL) and ossification of the nuchal ligament (ONL) in terms of incidence and size.

**Methods:**

This retrospective study evaluated 297 patients who underwent CT of the cervical spine (C-spine). Two radiologists worked in consensus. The incidence of OPLL from patients with and without ONL was compared using Chi Square tests. The mean lengths of ONL from patients with and without OPLL were compared using Student t-test. The correlations between the length of ONL and the presence of OPLL and between the length of ONL and the length of OPLL were analyzed with Pearson correlations.

**Results:**

We found that OPLL occurred more frequently in patients with ONL than in patients without ONL (odd ratio = 2.524, *p* = 0.037); however, the mean length of ONL did not differ significantly patients with and without OPLL (*p* = 0.874). We found no significant correlation between the length of ONL and the length of OPLL (*p* = 0.233).

**Conclusion:**

The presence of ONL was associated with the presence of OPLL. The length of OPLL and ONL showed no correlation.

## Introduction

Ossification of the posterior longitudinal ligament (OPLL) and ossification of the nuchal ligament (ONL) in the cervical spine are common types of ligament ossification of the spine [1]. OPLL can compress the spinal cord directly and may lead to neurologic symptoms, which require surgical intervention in severe cases [2]. The incidence of OPLL ranges from 0.1 to 2.8% and varies in relation to race and ethnicity [1,3,4]. OPLL is associated with other forms of paravertebral ligament ossification, such as diffuse idiopathic skeletal hyperostosis (DISH) and ossification of the ligamentum flavum [5,6]. ONL involves ossification in the soft tissue posterior to the spinous process of the cervical spine [7]. The incidence of ONL is significantly greater than that of OPLL, ranging from 4.5 to 11.3%, and it also varies in relation to race and ethnicity [1]. ONL forms as a result of trauma and chronic overload in the nuchal ligament; nevertheless, the effects of ONL on pain and cervical mobility are still unclear [7,8]. The purpose of this study was to investigate the association between OPLL and ONL in terms of incidence and size.

## Methods

### Patients

The Institutional Ethics Review Board at our hospital approved this study (KBSMC 2015-08-033-001). The requirement for informed consent was waived due to the retrospective study design. Two radiologists retrospectively evaluated 303 consecutive patients who underwent CT of the cervical spine (C-spine) between May 2005 and April 2015. The reason for C-spine CT examination was neck pain and mobility disturbance after trauma such as fall down, assault and car accident. We limited our study population to patients who underwent CT for trauma in order to avoid a selection bias of including a large fraction of patients with underlying spine pathology such as severe degenerative spondylosis. Exclusion criteria were a history of C-spine surgery (four cases), tumor of the bony structure of the C-spine (one case) and spondylitis of the C-spine (one case). Thus, a total of 297 patients were included in the study; there were 91 women and 206 men (mean age: 51.0 ± 18.5 years, range: 13–93 years).

### CT protocol

CT was performed with a 40- or 64-MDCT scanner (Brilliance Power, Philips Medical Systems, Andover, MA, USA). The scan coverage extended proximally from the base of the skull to T2 distally. The average scan length was about 240 cm. Contrast medium was not used. CT was performed with a 64 Χ 0.625 collimation, table feed of 38.7 mm/sec, and gantry rotation time of 0.5 seconds. The X-ray tube voltage was 120 kV, and amperage was 137 mAs. The weighted CT dose index was 8.9 mGy, in accordance with the protocol.

### Image analysis

**Fig 1:**
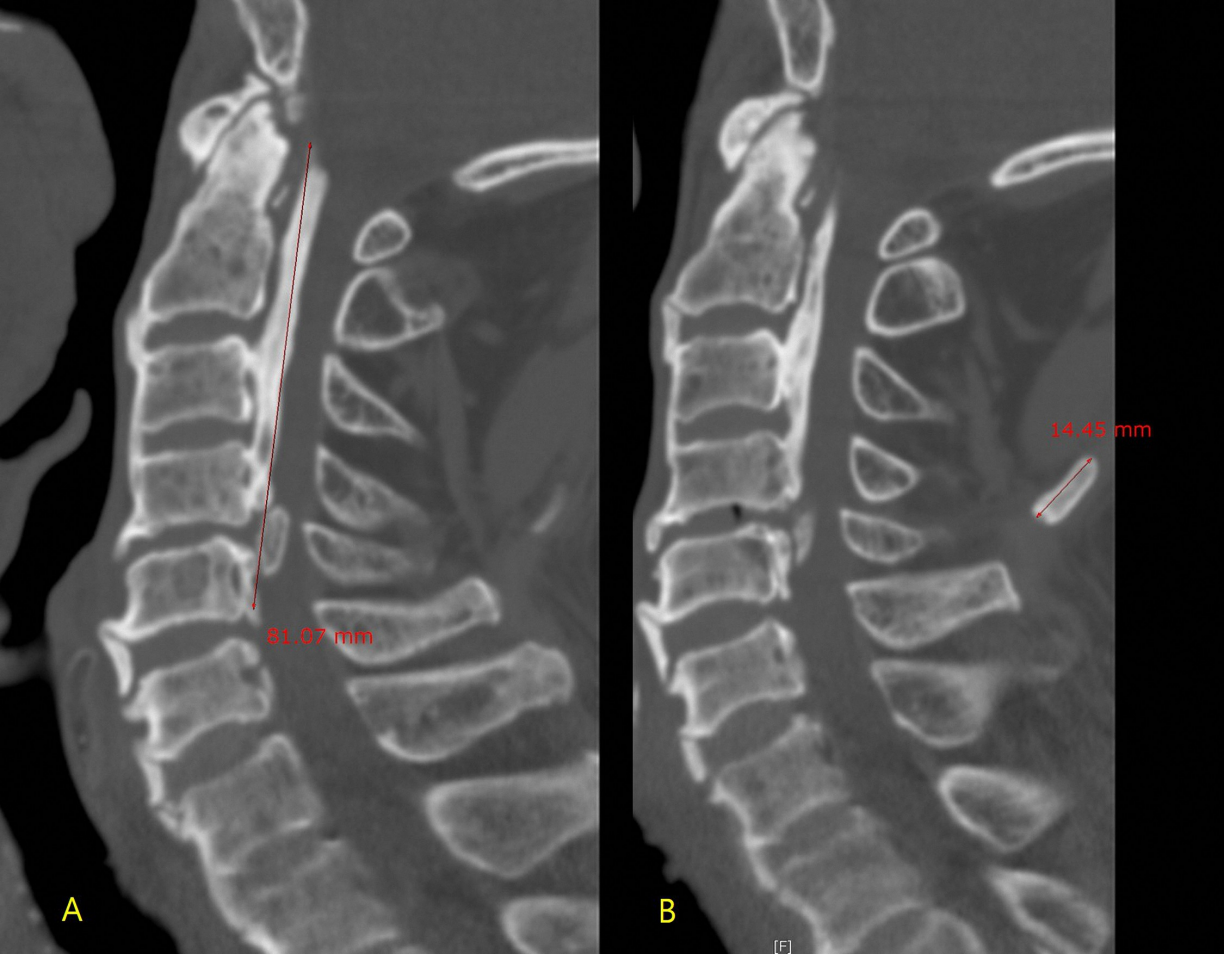
66-year-old man with neck pain and mobility disturbance after trauma. (A) The sagittal reconstructed CT image revealed ossification of the posterior longitudinal ligament extending from C1 to C5 and measuring 8.1cm along its entire length. (B) Another CT image showed ossification of the nuchal ligament extending from C4 to C5 and measuring 1.4cm along its entire length.

**Fig 2:**
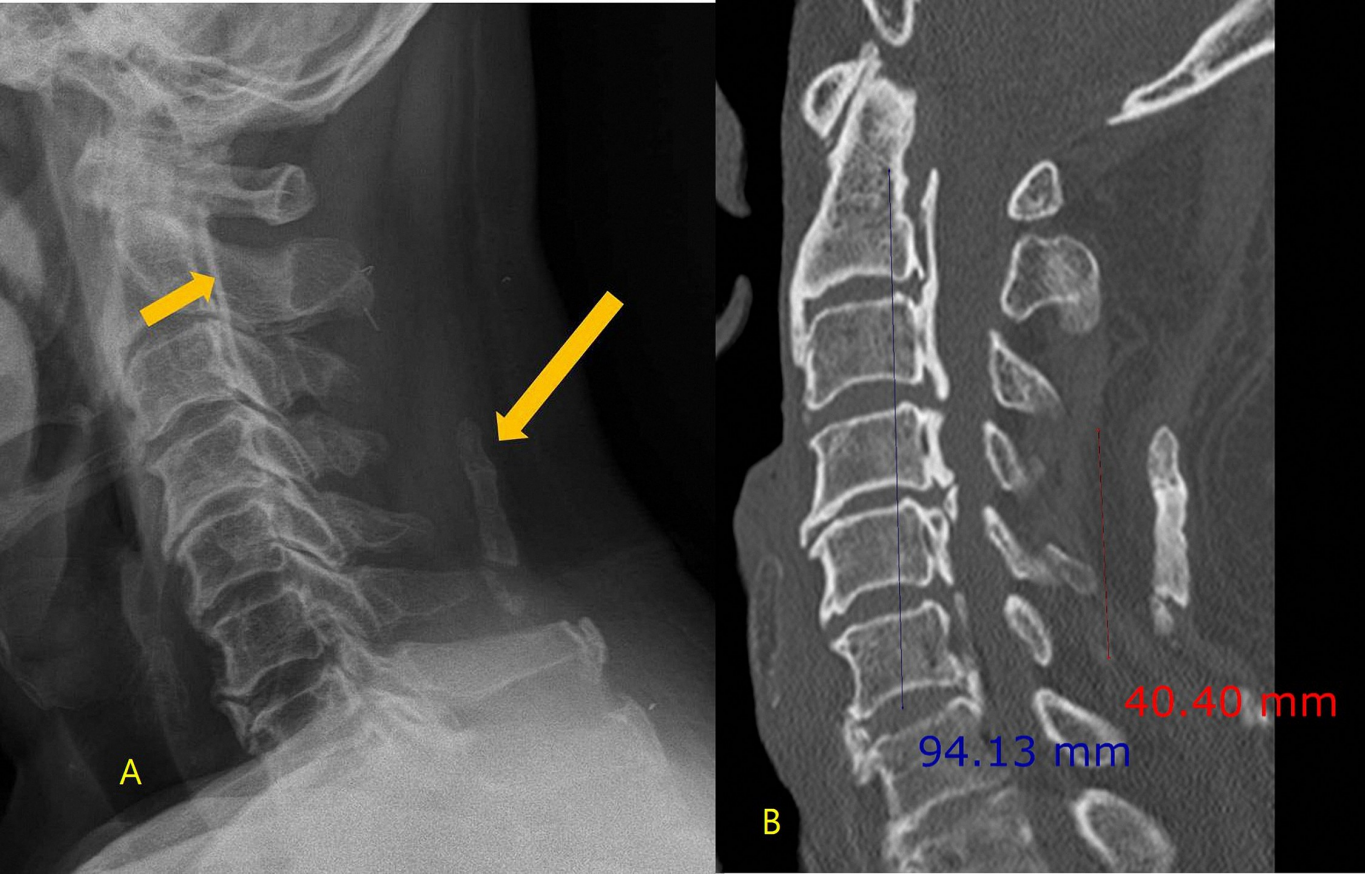
70-year-old man with neck pain after fall down. (A) Lateral radiograph of the cervical spine showing a linear increased density on the posterior surface of the vertebral bodies (short arrow) and ossification of the nuchal ligament (long arrow). (B) The sagittal reconstructed CT image revealed ossification of the posterior longitudinal ligament (long double arrow) and nuchal ligament (short double arrow).

**Fig 3:**
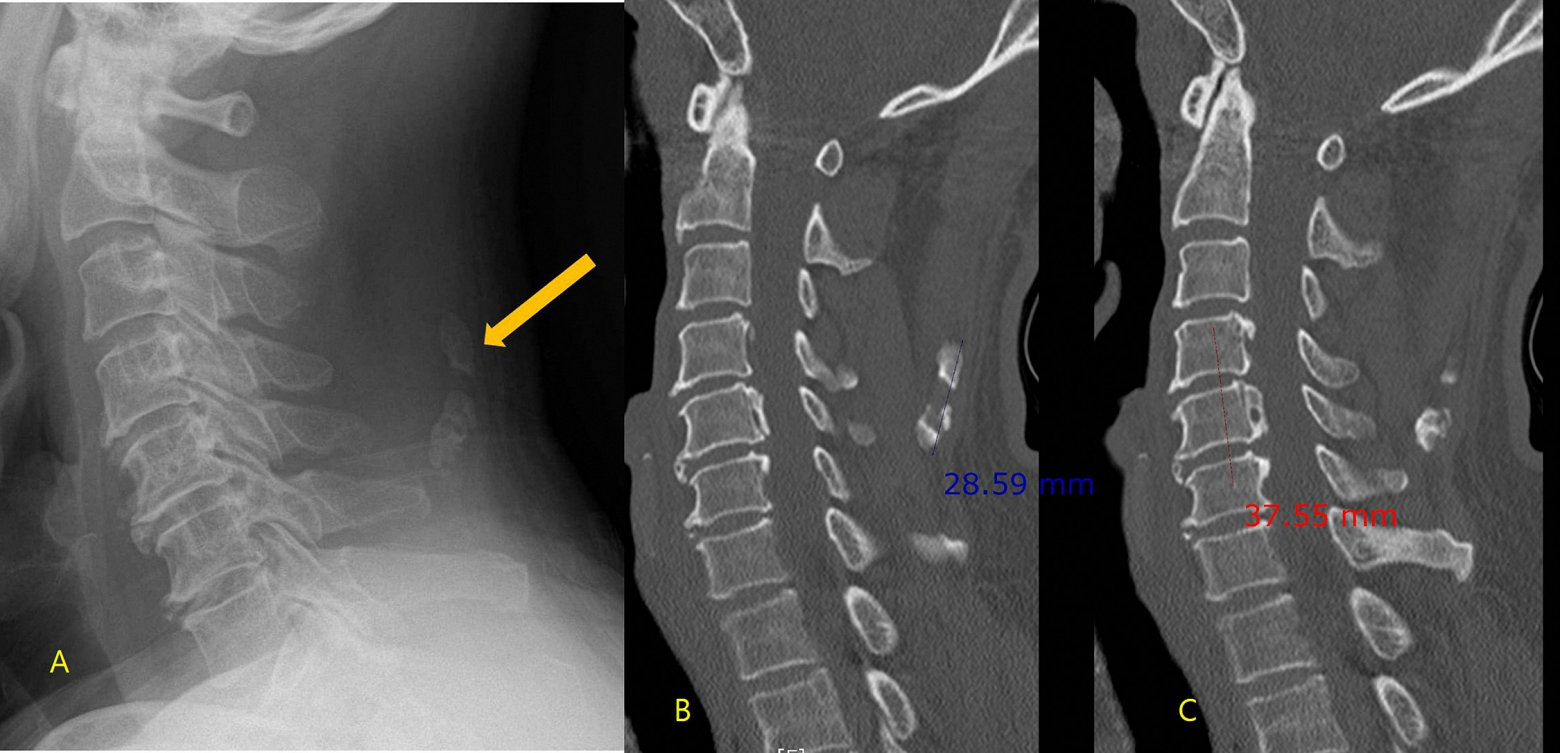
60-year-old man with neck discomfort and mobility disturbance after trauma. (A) Lateral radiograph of the cervical spine showing ossification of the nuchal ligament (arrow). However, ossification of the posterior longitudinal ligament was not clearly visible. (B) The sagittal reconstructed CT image revealed ossification of the nuchal ligament extending from C4 to C5 and measuring 2.9cm along its entire length. (C) Another CT slice showing ossification of the posterior longitudinal ligament extending from C4 to C6 and measuring 3.8cm along its entire length.

C-spine CT was evaluated with a picture archiving and communication systems (PACS, Infinitt 3.0, Seoul, Korea) by two fellowship-trained musculoskeletal radiologists with 14 and 9 years of experience. The two radiologists worked in consensus and were blinded to the previous radiologic reports. First, they evaluated whether OPLL or ONL was present. A diagnosis of OPLL was made if the CT demonstrated an ossified mass along the posterior longitudinal ligament of the C-spine [8]. A diagnosis of ONL was made if the CT demonstrated a radiopaque density in the soft tissue posterior to the spinous processes of the C-spine [7]. Then they measured the length from the superior end of each ossification (OPLL and ONL) to the inferior end (Figs 1–3) by consensus based on a sagittal reconstruction image of the C-spine. If there was segmental OPLL or ONL, the regions were measured in aggregate. Two radiologists worked together to make diagnoses and to make decision where to measure.

### Statistical analysis

The total incidence of OPLL and ONL was calculated. The mean lengths of OPLL and ONL were also calculated. The incidence of OPLL from patients with and without ONL was analyzed at odd ratios. The mean length of ONL from patients with and without OPLL and the mean length of OPLL from patients with and without ONL were compared using Student t-test. Correlations between the length of ONL (each differing length of ONL) and the presence of OPLL and between the length of ONL and the length of OPLL (each differing length of OPLL) were evaluated with Pearson correlations. A correlation coefficient (R) between 0.1 and 0.3 was interpreted as a weak correlation; between 0.3 and 0.7, as a moderate correlation; between 0.7 and 0.9, as a strong correlation; and above 0.9, as a very strong correlation [9]. *P*-values < 0.05 were considered statistically significant. Statistical analyses were performed with PASW software version 18.0 (IBM, Armonk, NY, USA).

## Results

The incidences of OPLL and ONL are summarized in Table 1. A total of 23 (8%) of the 297 patients had OPLL. Of these 23 patients, 10 (43%) had OPLL and ONL, and 13 (57%) had OPLL, but not ONL. A total of 74 (32%) of the 297 patients had ONL. In the patients with ONL, 10 patients (13.5%) were identified as having OPLL, while in the patients without ONL, 13 patients (5.8%) were identified as having OPLL. OPLL occurred more frequently in patients with ONL than in patients without ONL, which was significantly different (*p* = 0.037). The odds ratio was 2.52 (95% CI 1.06–6.03). The mean lengths of OPLL and ONL are summarized in Table 2. The mean length of OPLL was about 38 mm, and the mean length of ONL was about 15 mm. The mean length of ONL did not differ significantly in patients with and without OPLL (*p* = 0.874), and also the mean length of OPLL did not differ significantly in patients with and without ONL (*p* = 0.585). Table 3 shows the correlation coefficients between the length of ONL and presence of OPLL and between the length of ONL and length of OPLL. The length of ONL was not significantly correlated with the presence and the length of OPLL (*p* = 0.874 and 0.233, respectively).

**Table 1:** Incidence of OPLL and ONL.

	ONL (+)	ONL(-)	Total
OPLL(+)	10 (13.51%)	13 (5.83%)	23 (7.77%)
OPLL(-)	64 (86.49%)	210 (94.17%)	274 (92.23%)
Total	74 (100%)	223 (100%)	297 (100%)

Odds ratio = 2.524 (increased risk for having OPLL in the presence of ONL than absence of ONL)

95% confidence interval = 1.06–6.03

*P* value = 0.037

OPLL = Ossification of the posterior longitudinal ligament

ONL = Ossification of the nuchal ligament

**Table 2:** Mean length of OPLL and ONL (mm).

	OPLL	ONL
OPLL(+), ONL(+)	41.60 (±21.73)	15.80 (±10.02)
OPLL(+), ONL(-)	35.85 (±26.60)	
OPLL (-), ONL(+)		15.28 (±9.50)
Total	38.35 (±24.24)	15.35 (±9.50)
*P* value	0.585	0.874

OPLL = Ossification of the posterior longitudinal ligament

ONL = Ossification of the nuchal ligament

Note: Data in parentheses represent standard deviation

**Table 3:** Pearson correlation coefficients (CCs) between the length of ONL and the presence of OPLL and between the length of ONL and length of OPLL.

	Correlation coefficients	*P* value
Presence of OPLL and length of ONL	0.019	0.874
Length of OPLL and ONL	0.415	0.233

ONL = Ossification of the nuchal ligament

OPLL = Ossification of the posterior longitudinal ligament

Note: The strength of correlation was characterized as follows: weak correlation (0.1 < CCs ≤ 0.3), moderate correlation (0.3 < CCs ≤ 0.7), relatively high correlation (0.7 < CCs ≤ 0.9), very high correlation (0.9 < CCs).

## Discussion

The pathogenesis of OPLL is still unknown. Many studies have indicated that OPLL cells have several osteoblastic phenotypes that differ from those of normal spinal ligament cells [10,11]. OPLL is common in the Asian population, so genetic factors are considered to play an important role [12]. The collagen 6A1 gene is associated with OPLL, and bone morphogenic protein-2 (BMP-2) and TGF-β appear to be important factors in OPLL formation [13–15]. Clinically non-insulin-dependent diabetes mellitus, higher bone mineral density, and lifestyle including a high-salt diet are suggested to increase the risk of developing OPLL [16–18]. Mechanical stress on the spinal ligaments has been investigated as a cause of OPLL [19]. OPLL is twice as common in men as in women, and it is more common among older patients in their fifties and sixties [20]. ONL is also more common in men (1.5 times) and shows the highest incidence in patients older than 69 years [8]. The nuchal ligament (NL) is a triangular fibrous membrane that extends from the external occipital protuberance to the spinous process of C7 [21]. The NL is thought to be an important structure for maintaining lordotic alignment [22–24]. Trauma and chronic overload in the cervical spinal ligament may cause ONL, which is closely associated with cervical spondylosis [8,21–25].

ONL may be a spinal ligament ossification syndrome, like ossification of the ligamentum flavum, ossification of the anterior longitudinal ligament or ossification of the posterior longitudinal ligament, and, therefore, ONL could coexist with disorder of ossification of other spinal ligaments [18]. In our study, the incidence of OPLL in patients with ONL (13.5%) was significantly higher than in patients without ONL (5.8%). A few studies have reported the relationship between OPLL and ONL. Kim *et al* reported that the incidence of OPLL was almost 4 times greater in patients with ONL than without ONL. Compared to the incidence of OPLL in patients with ONL (64.7%) and without ONL (16.1%) reported by Kim et al. [26] however, the incidence of OPLL in patients with and without ONL was considerably lower in our study. Wang *et al* reported that ONL is twice as common in patients with OPLL as in patients without OPLL and multilevel ONL occurs mostly in patients with OPLL [8]. Yoshii et al reported that ossification index of OPLL increased as the levels of ONL increased [27]. In our study, however, the mean length of ONL did not differ significantly in patients with and without OPLL, and the mean length of OPLL did not differ significantly in patients with and without ONL. Moreover, the length of ONL was moderately but not significantly correlated with the length of OPLL. Patient selection may be the major reason for these differences. Kim *et al* and Wang *et al* had focused on patients with degenerative cervical spondylosis. The study of Yoshii et al was based on a study of 233 patients who had OPLL. However, our study was based on trauma patients because we wanted to know true incidence of the ONL and OPLL without bias of degenerative change and clinical manifestation of the spinal stenosis.

ONL is easier to see on plain radiography than OPLL because ONL is easily distinguished from fat and soft tissue while OPLL is difficult to perceive when overlapping bony structures are present (Fig 3). Therefore, clinicians observing ONL should strive to find OPLLs and should consider additional studies such as CT scans of cervical spine; however, a large ONL was not indicative of presence of OPLL.

This study had several limitations. First, this was a retrospective study, so it may be biased in regards to patient selection. There might be more cases of cervical spine degeneration or OPLL, because patients with walking disorders can easily fall over. Second, measuring the length of OPLL and ONL might not have been appropriate because some cases of OPLL or ONL were discontinuous in shape. The shape of OPLL can be continuous, segmental, or localized [28], so we may have overestimated the length of ossification in patients with segmental ossification. Third, risk factors such as age and gender may be confounding factors for OPLL and ONL, and in our study, the results were not adjusted to account for these variables. As previously discussed, the incidence of ossification of the spinal ligament is almost two times greater in males than in females, and it increases with age, being more common in people older than 50 years [8,20]. Fourth, we did not take into account the individual differences in physique in the study.

In conclusion, patients in a random trauma population are 2.5 times more likely to have OPLL when ONL is present. However, the length of ONL was not related to the incidence of OPLL and there was no correlation between the length of ONL and OPLL. The incidentally detected ONL, which can easily be seen on plain radiography, may predict OPLL, which is difficult to see on plain radiography.

## Main Points

1. Patients in a random trauma population are 2.5 times more likely to have OPLL when ONL is present.
2. The length of ONL was not related to the incidence of OPLL and there was no correlation between the length of ONL and OPLL.
3. The incidentally detected ONL, which can easily be seen on plain radiography, may predict OPLL, which is difficult to see on plain radiography.

## Supporting information

S1 Data Set Anonymized data. (XLSX)
